# Meetings of Societies

**Published:** 1948

**Authors:** 


### Meetings of Societies

BRITISH MEDICAL ASSOCIATION (BATH, BRISTOL & SOMERSET BRANCH). *President*, J. R. Nicholson-Lailey, F.R.C.S. Programme for 1949: January 26th, 1949, Taunton; February 23rd, 1949, Bath; March 30th, 1949, Bristol; May 25th, 1949, Weston-super-Mare.

DEVON AND EXETER MEDICO-CHIRURGICAL SOCIETY.

THE first meeting of the session was held on October 6th, 1948. Dr. Horace Evans spoke of hypertensive disease with special reference to surgical treatment: some account of hi address appears on page 112.

The second meeting was held at the Royal Devon and Exeter Hospital on November 18th. Dr. Anthony Daly spoke on the treatment of thyrotoxicosis, a subject on which we have published two papers this year.

SOCIETY OF ANAESTHETISTS OF THE SOUTH-WESTERN REGION

THE second annual general meeting of the Society was held at Bristol on October 29th and 30th, 1948. Fifty-seven members were present. Dr. John Gillies of Edinburgh, President of the Association of Anaesthetists of Great Britain and Ireland, gave an address entitled: " **Spinal Analgesia—its present status** ". Other papers were read by Mr. Geoffrey FitzGibbon, on " **Problems of Anaesthesia for Plastic Surgery** "; by Dr. T. B. P. Wilton on " **Anaesthesia for Thoracoplasty** ", and by Dr. R. F. Woolmer on " **The Explosion Risk** ". Practical demonstrations were also given at the Bristol Royal Hospital and at the Thoracic Unit, Frenchay.

Attention is drawn to the announcement on page 113, relating to future arrangements.

